# Impact of mitral regurgitation severity on thromboembolic risk in atrial fibrillation: a comprehensive systematic review and meta-analysis

**DOI:** 10.1007/s10554-025-03487-7

**Published:** 2025-08-08

**Authors:** Adil Salihu, Georgios Tzimas, Pierre Monney, Niccolo Maurizi, Ioannis Skalidis, Dimitri Arangalage, Denise Auberson, Sarah Hugelshofer, Cheryl Teres, Olivier Muller, Henri Lu, Panagiotis Antiochos

**Affiliations:** Service of Cardiology, Lausanne University Hospital and University of Lausanne, Lausanne, 1011 Switzerland

**Keywords:** Atrial fibrillation, Mitral regurgitation, Thromboembolic event, Left atrial thrombus

## Abstract

Atrial fibrillation (AF) is associated with a heightened risk of thromboembolic events (TE). Whether severe mitral regurgitation (MR) has a protective effect against thrombus formation in AF remains unclear. We aimed to evaluate the impact of MR severity on thromboembolic risk in patients with AF. This systematic review with meta-analysis included data from eight studies, from 1998 to 2022. All studies reported the prevalence of TE according to MR severity, in patients with non-valvular AF, defined as the absence of prosthetic/repaired mitral valve or moderate or more severe mitral stenosis. TE was defined as stroke, transient ischemic attack, another arterial embolic event, or presence of thrombus in the left atrium. Eight studies with a total of 21,821 patients (54% male, mean age 70 ± 11 years) were identified. The overall prevalence of TE was 6.0%. No significant difference was observed in the prevalence of TE based on MR severity (no or mild MR: 5.9%, moderate or more severe MR: 6.8%, odds ratio: 1.11, 95% confidence interval: 0.77–1.73, *p* = 0.64). When specifically examining studies that exclusively reported either embolic or thrombotic events, there was no statistically significant difference in the incidence rates of events observed across the different categories of MR severity. Our analysis found no significant association between MR severity and the risk for TE, in patients with non-valvular AF. This suggests that MR may not be a determining factor for thromboembolic risk in this population.

**Clinical Trial registration (PROSPERO)** CRD42024504403.

**Graphical abstract:**

AF: Atrial fibrillation, MR: Mitral regurgitation

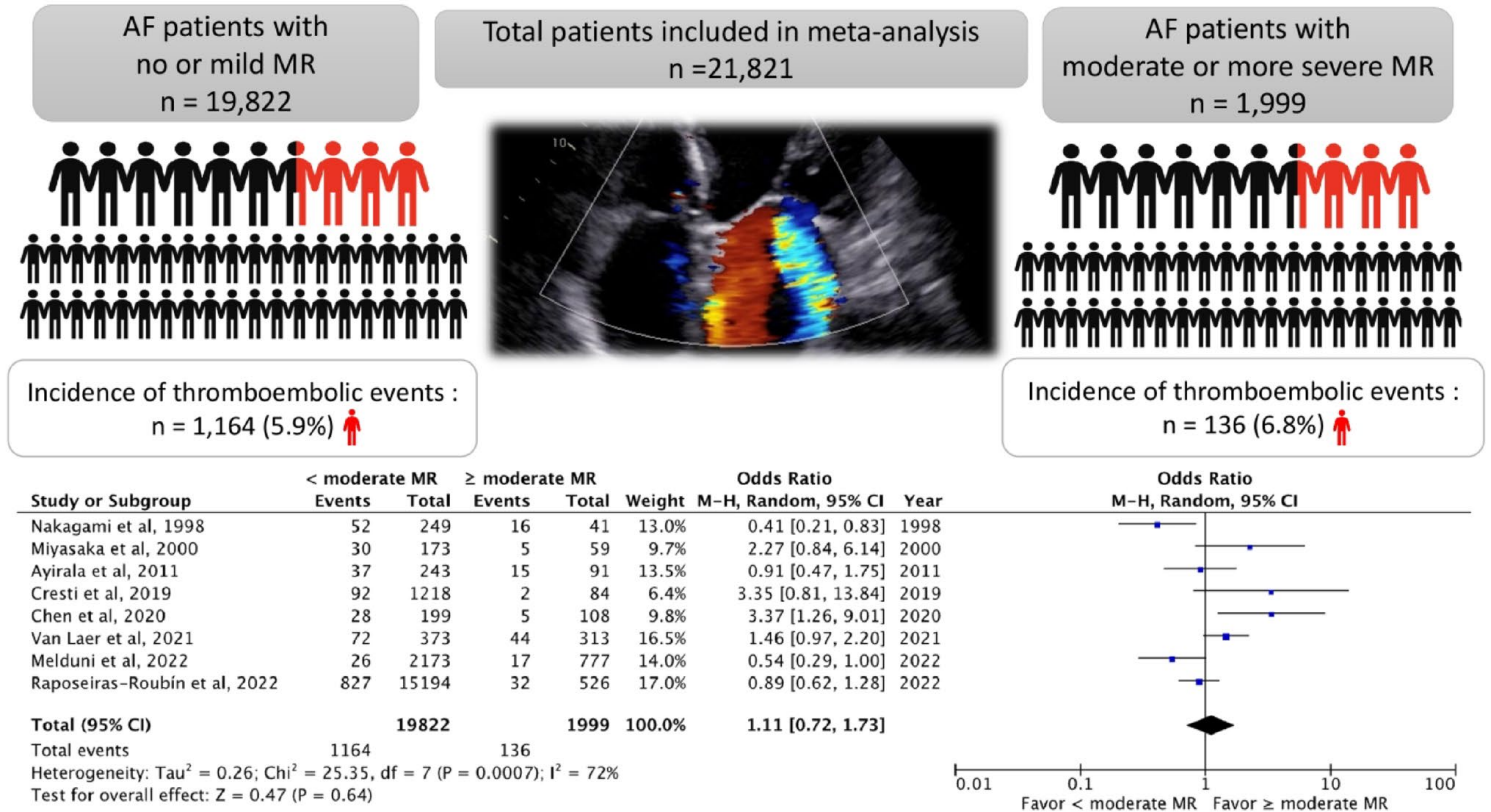

**Supplementary Information:**

The online version contains supplementary material available at 10.1007/s10554-025-03487-7.

## Introduction

Atrial fibrillation (AF), the most common sustained cardiac arrhythmia, significantly increases the risk of thromboembolic events (TE), including ischemic stroke, by promoting a hypercoagulable state [1–3]. This risk is traditionally quantified using the CHA_2_DS_2_-VASc score, which contains a range of clinical factors such as congestive heart failure, hypertension, age, diabetes, history of stroke, vascular disease, and sex. While this scoring system provides a solid framework for risk stratification and therapeutic decision-making, it highlights that the thromboembolic risk in AF is not solely a consequence of the arrhythmia itself but further dictated by a spectrum of coexisting clinical conditions [2]. This multifactorial risk prediction prompts a critical examination of other potential modulators of thromboembolic risk in AF patients, beyond those encapsulated by the CHA_2_DS_2_-VASc score.

Mitral regurgitation (MR) has emerged as a condition of particular interest. Traditionally, severe MR has been hypothesized to play a protective role against thrombus formation in the left atrium. The rationale is that the significant regurgitative jet “washes out” the left atrium and, more specifically, the left atrial appendage (LAA), potentially reducing the likelihood of left atrial thrombus (LAT) formation. The relationship between MR severity and the incidence of LAT has been particularly studied in the context of severe rheumatic MR. In such cases, a noteworthy inverse correlation has been documented, indicating that more severe MR could be associated with a lower occurrence of LAT [4–6]. Yet, this observed relationship has not been consistently replicated in other studies, leaving its validity in question to this day. Moreover, the role of MR severity as a factor associated with thromboembolic risk is likewise disputed among patients with non-rheumatic, non-valvular AF, where existing studies have reached conflicting conclusions.

To address these discrepancies, we conducted a comprehensive systematic review and meta-analysis of all relevant literature to date, aiming to accurately assess the association between MR severity and thromboembolic risk, among patients with non-valvular AF.

## Materials and methods

This systematic review with meta-analysis followed a prespecified study protocol registered on the PROSPERO international prospective register of systematic reviews (Risk of thromboembolic events according to MR severity in patients with AF: a systematic review and meta-analysis; CRD42024504403). Results are reported according to the Preferred Reporting Items for Systematic Reviews and Meta-Analyses (PRISMA) 2020 statement (Supplemental Table 1).

### Literature search and selection criteria

A systematic review of the literature was performed using the online databases PubMed/MEDLINE (Medical Literature Analysis and Retrieval System Online), Embase (Excerpta Medica Database) and the Cochrane Library, from inception to December 31 st, 2024, using the following search strategy: (atrial fibrillation) AND (mitral regurgitation) AND ((thrombus) OR (thromboembolic event) OR (stroke) OR (embol*)) (Supplemental Table 2). Furthermore, the references of all selected studies were thoroughly examined to identify additional relevant articles.

### Eligibility criteria and selection process

We included all studies reporting the prevalence of TE according to MR severity in patients with AF. Studies reported in case reports, case series, or published abstracts with limited data were not considered eligible for inclusion.We excluded non-human and non-English studies, as well as studies including patients with rheumatic mitral disease, at least moderate mitral stenosis, a mechanical or biological mitral prosthesis, or previous mitral valve repair. Ethical approval was not required, as this was a study-level meta-analysis of published data.

Two reviewers, A.S. and P.A., independently assessed the articles for inclusion based on their titles and abstracts, using the Covidence systematic review software (Veritas Health Innovation, Melbourne, Australia). Whenever discrepancies arose, a third reviewer, H.L., was consulted to resolve them.

### Data extraction and quality assessment

The following information was extracted from each study: first author’s name, year of publication, patients’ inclusion dates, type of study, number and baseline characteristics of patients, type of AF (paroxysmal, persistent, or permanent, when available), embolic events and LAT, MR severity, and modality of quantification (mild, moderate, severe, whenever information was available).

The methodological quality of each study included in the analysis was assessed using the Newcastle Ottawa Scale (NOS). This scale integrates the evaluation of three categorical data sets, with a total possible score of nine points. The primary component of this tool is allocated up to four stars, focusing predominantly on the methodological integrity of each primary study. An additional aspect of the tool, which can receive up to three stars, pertains primarily to the comparability across different studies. The final element, rated up to three stars, is employed to scrutinize the outcomes and statistical methodologies of each original study. The assessment criteria define studies scoring 7 points or higher as “good”, those achieving 2 to 6 points as “fair”, and studies with 1 point or less as being of “poor” quality.

### Outcomes

The primary outcome was the prevalence of TE according to MR severity. MR severity was graded based on objective measurements made according to international guidelines whenever available, or on visual qualitative assessment, as reported by the authors. In patients with AF, a TE was defined as a combined endpoint including: (i) stroke or transient ischemic attack (TIA), (ii) peripheral embolism other than stroke or TIA, and (iii) thrombus formation in the LA including the LAA.

Secondary outcomes included the individual prevalence of (i) LAT and (ii) embolic events, according to MR severity. An embolic event was defined as an ischemic stroke, TIA, or any peripheral embolic event within the arterial circulation. A thrombotic event was defined as the presence of LAT, as confirmed by transoephageal echocardiography (TEE).

Patients were categorized as having moderate or more severe MR (moderate, moderate-to-severe, severe) versus less than moderate MR (no MR, mild, mild-to-moderate) for the purposes of the meta-analysis [7, 8]. 

### Statistical analyses

Data were summarized using descriptive statistics with means ± standard deviation (SD) for continuous variables, and frequencies with percentages for dichotomous variables. Meta-analyses were performed by combining the results of the published incidence of the predetermined outcomes. The odds ratios (ORs) and their 95% confidence intervals (CIs) were used as summary statistics. The I^2^ statistic was used to estimate the percentage of total variation across studies due to heterogeneity rather than chance: intervals of < 25%, 25–50%, and > 50% were used to classify heterogeneity as low, moderate, and high [9]. The Mantel-Haenszel method within a random-effects model framework was used to estimate pooled outcomes, accounting for population diversity and methodological heterogeneity across studies. Additionally, pooled incidences of TE for each MR severity group were calculated using a random-effects meta-analysis of single proportions, employing a Freeman-Tukey double arcsine transformation. These pooled incidences are reported with their respective 95% Cis. All p-values were two-sided, and a threshold of < 0.05 was considered statistically significant. Finally, a “leave-one‐out” sensitivity analysis was conducted by sequentially excluding each individual study from the pooled data to assess the robustness of our overall findings. The analyses were performed using Review Manager (RevMan, Version 5.4.1 The Cochrane Collaboration, Copenhagen, Denmark) and Stata Statistical Software, Release 17.0 (StataCorp. 2021. College Station, TX, StataCorp LLC, USA). Publication bias was assessed through visual inspection of funnel plots for asymmetry.

## Results

### Article selection

**Fig. 1:**
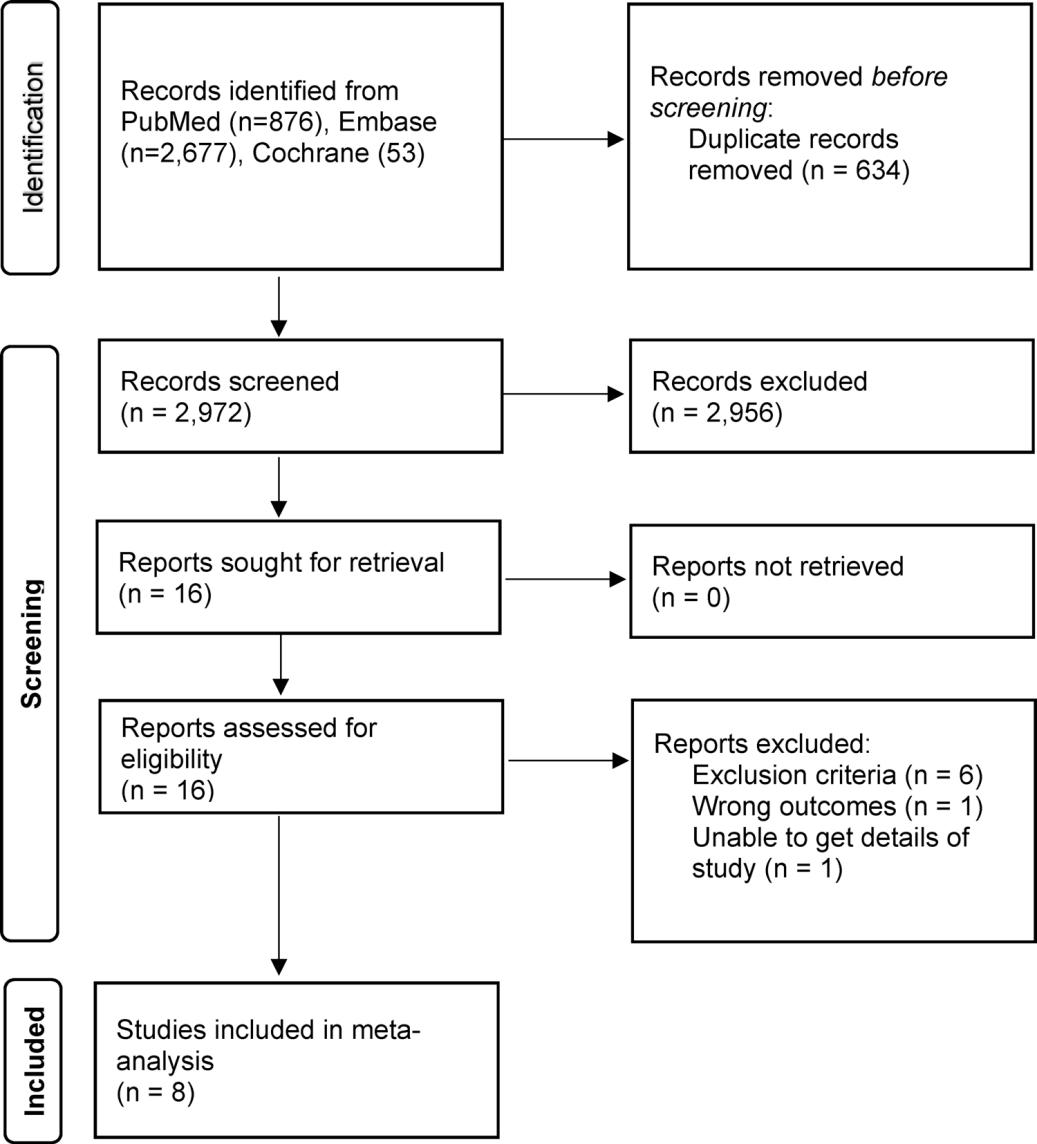
Prisma flow diagram

In the preliminary search, a total of 3,606 articles was identified: 876 from PubMed, 2,677 from Embase, and 53 from the Cochrane Library, as illustrated in the PRISMA flowchart (Fig. 1). Upon further inspection, 634 articles were determined to be duplicates, leaving 2,972 articles for comprehensive assessment. Of those, 2,956 were excluded after title and abstract screening. The remaining 16 articles were assessed for eligibility at full-text level, with eight more being excluded, due to predefined exclusion criteria or inability to obtain reported outcomes. Ultimately, eight studies met the predefined inclusion criteria and were included in the final analysis [10–17].

### Quality assessment

The quality of the eight included studies was moderate based on the NOS tool assessment. Details are presented in Supplemental Table 3. Funnel plot analysis assessing publication bias is presented in Supplemental Fig. 1.

### Characteristics of the overall cohort

The eight studies included a total of 21,821 patients (19,822, or 91% with less-than-moderate MR and 1,999, or 9% with moderate or more severe MR). All studies were retrospective in design. The characteristics of the studies are presented in Table 1.

**Table 1:** Baseline characteristics of studies included evaluating the prevalence of thromboembolic event according to MR severity

Authors	Year of inclusion	Design	Total number of patients	Paroxysmal AF (in %)	Mitral regurgitation	
					< moderate	≥ moderate
Nakagami et al. (1998) [10]	1987-1995	Retrospective	290	NR	249	41
Miyasaka et al. (2000) [11]	1996-1997	Retrospective	232	99 (43)	175	59
Ayirala et al (2011) [12]	2003-2010	Retrospective	334	NR	243	91
Cresti et al. (2019) [13]	NR	Retrospective	1302	1024 (79)	1218	84
Chen et al. (2020) [14]	2013-2014	Retrospective	307	NR	199	108
Van Laer et al. (2021) [15]	2013-2018	Retrospective	686	306 (45)	373	313
Melduni et al. (2022) [16]	2000-2012	Retrospective	2950	1093 (37)	2153	777
Raposeiras-Roubín et al. (2022) [17]	2014-2018	Retrospective	15720	2645 (17)	15194	526

AF: atrial fibrillation, MR: mitral regurgitation

Baseline characteristics of the study population stratified according to MR severity are presented in Table 2. Patients with moderate or more severe MR were significantly older, were more often male, and had a lower body mass index. Additionally, they had a higher prevalence of coronary artery disease and peripheral artery disease. The CHA_2_DS_2_-VASc score was significantly higher in patients with moderate or more severe MR, although the prevalence of paroxysmal AF and the utilization of anticoagulation therapy were comparable between the two groups. Finally, mean left ventricular ejection fraction (LVEF) was lower in patients with moderate or more severe MR (Table 3).

**Table 2:** Baseline characteristics and procedural data with comparison according to MR severity

Characteristics	Number of studies (references)	< moderate MR	≥ moderate MR	*p*-value	Total
Clinical					
Age (in years)	[11, 13, 15–17]	69 ± 11	73 ± 11	p = 0.005	70 ± 11
Male gender, n (%)	[11, 13, 15–17]	10,234 (53)	966 (55)	*p* < 0.001	11,200 (54)
BMI (in kg/m^2^)	[15, 16]	31 ± 7	29 ± 6	*p* < 0.001	30 ± 7
HTA, n (%)	[11, 13, 15–17]	13,397(70)	1164 (66)	*p* = 0.25	14,561 (70)
DM, n (%)	[11, 15–17]	5096 (19)	753 (21)	*p* = 0.50	5849 (30)
Prior TIA/stroke, n (%)	[15–17]	1254 (7)	170 (11)	*p* = 0.81	1424 (7)
CAD, n (%)	[16, 17]	2098 (12)	303 (23)	*p* < 0.001	2401 (13)
PAD, n (%)	[15–17]	914 (5)	260 (16)	*p* = 0.02	1184(6)
Anticogulation, n (%)	[11, 13, 15–17]	14,665 (77)	1388 (79)	*p* = 0.07	16,053 (77)
Paroxysmal atrial fibrillation, n (%)	[11, 13, 15–17]	4645 (24)	537 (31)	*p* = 0.37	5182 (25)
CHA_2_DS_2_-VASc score	[16, 17]	3.2 ± 1,5	3.6 ± 1.6	*p* < 0.001	3,2 ± 1,5
Echocardiographical					
LVEF (in %)	[9, 11, 12]	52 ± 13	49 ± 15	*p* < 0.001	51 ± 14

BMI: body mass index, CAD: coronary artery disease, DM: diabetes mellitus, HTA: arterial hypertension, LVEF: left ventricular ejection fraction, PAD: peripheral artery disease, TIA: transient ischemic attack

**Table 3:** Prevalence of thromboembolic event according to MR severity

Authors	Total number ofpatients	Embolic events		Left atrial thrombus	
		< moderate MR (n/%)	≥ moderate MR (n/%)	< moderate MR (n/%)	≥ moderate MR (n/%)
Nakagami et al. (1998) [10]	290	52 (21)	16 (39)	NR	NR
Miyasaka et al. (2000) [11]	232	30 (17)	5 (8)	NR	NR
Ayirala et al (2011) [12]	334	NR	NR	37 (15)	15 (16)
Cresti et al. (2019) [13]	1302	NR	NR	92 (8)	2 (2)
Chen et al. (2020) [14]	307	NR	NR	28 (14)	5 (5)
Van Laer et al. (2021) [15]	686	NR	NR	72 (19)	44 (14)
Melduni et al. (2022) [16]	2950	NR	NR	26 (12)	17 (22)
Raposeiras-Roubín et al. (2022) [17]	15720	827 (5)	32 (6)	NR	NR

AF: atrial fibrillation, MR: mitral regurgitation

### Definition of mitral regurgitation

There was substantial variability in the criteria that were used to define the severity of MR. Several studies employed a semi-quantitative approach, assessing the distance of the MR jet relative to the size of the LA [10, 11, 16]. In contrast, other articles utilized a quantitative evaluation based on the Proximal Isovelocity Surface Area (PISA) method [10, 12, 15]. Some studies, however, did not provide specific details regarding their evaluation methodology, merely indicating adherence to contemporary guidelines [13, 14, 17].

The severity of MR was categorized as absent, mild, moderate, or severe in all studies. In five studies [10–12, 14, 17], moderate and severe MR were combined, while in one study [13], moderate and mild MR were grouped together. In this particular study, and for the purposes of the analysis, the inability to distinguish between these two subgroups led to their classification within the “no or mild MR” group. In the remaining two studies [15, 16], moderate and severe MR were distinctly categorized.

### Primary outcome

**Fig. 2:**
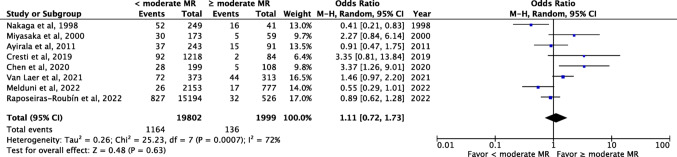
Pooled risk of thromboembolic events in patients with AF according to mitral regurgitation severity. AF: atrial fibrillation, MR: mitral regurgitation

The overall crude incidence of TE across all studies was 6.0%. Meta-analysis of proportions yielded a pooled incidence of TE in the ‘no or mild MR’ group of 11.2% (95% CI: 6.9% − 16.3%; I²= 98%). For the ‘moderate or more severe MR’ group, the pooled incidence of TE was 9.1% (95% CI: 4.4% − 15.2%; I²= 93%). The overall crude rates reported in the individual studies for these groups were 5.9% and 6.8%, respectively. The meta-analysis comparing the two groups yielded a pooled OR of 1.11 (95% CI: 0.72, 1.73, *p* = 0.64), indicating no significant difference in the odds of TE based on MR severity (Fig. 2). The I² for the OR meta-analysis was 72%.

One study [10] showed a protective association with lower risk for the “no or mild” MR group (in comparison with the “moderate or more severe” MR group). Conversely, one study [14] showed a relative protective association with lower risk for the “moderate or more severe” MR group against TE. The remaining six studies showed a neutral association of moderate or more severe MR regarding the risk of TE [11–13, 15–17].

In sensitivity analyses, omitting one study at a time, the pooled OR ranged from 0.97 to 1.26, with confidence intervals that consistently included 1, indicating no statistical significance, and with I² values ranging from 67 to 76%. No single study significantly altered the effect estimate or the overall conclusions. A summary of this analysis is presented in Supplemental Table 4. More specifically, when the study conducted by Raposeira-Roubin et al., which included 15,720 patients (72% of all patients who were included), was excluded from the meta-analysis, results remained consistent and no significant difference was observed between groups. Similarly, when the study by Cresti et al., which proposed a classification different from that of other studies, was excluded, the results remained consistent, and no significant difference was observed between groups.

### Secondary outcomes

**Fig. 3:**
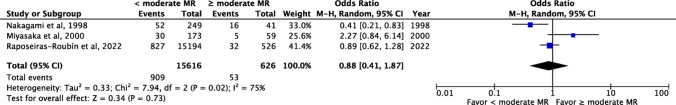
Pooled risk of embolic events in patients with AF according to mitral regurgitation severity. AF: atrial fibrillation, MR: mitral regurgitation

Among the three studies reporting only embolic events [10, 11, 17] the meta-analysis of proportions yielded a pooled incidence in the ‘no or mild MR’ group of 13.6% (95% CI: 3.8% – 27.0%; I²=97%). For the ‘moderate or more severe MR’ group, the pooled incidence of embolic events was 14.9% (95% CI: 2.4% – 34.4%; I²=93%). The comparative analysis between these groups showed no significant difference in the odds of embolic events (OR, 0.88, 95% CI, 0.41, 1.87, *p* = 0.93; I² for OR meta-analysis = 75%), as presented in Fig. 3. It is worth noting that the definition of an embolic event varied among studies, including solely stroke [10], a composite of stroke and TIA [17], or a broader composite that included stroke, TIA, and peripheral embolisms excluding stroke [11].

**Fig. 4:**
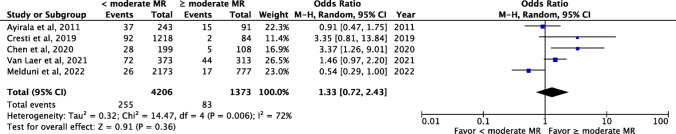
Pooled risk of thrombotic events in patient with AF according to mitral regurgitation severity. AF: atrial fibrillation, MR: mitral regurgitation

Five studies reported the presence of LAT [12–16], with TEE being the exclusive method of assessment in all cases, typically prior to external cardiac cardioversion [12–16]. The meta-analysis of proportions yielded a pooled incidence of LAT in the ‘no or mild MR’ group of 10.1% (95% CI: 3.4%– 19.8%; I²=98%). In the ‘moderate or more severe MR’ group, the pooled incidence of LAT was 6.8% (95% CI: 1.8%– 14.4%; I²=94%). The comparative analysis between these groups showed no significant difference in the odds of LAT (OR, 1.33, 95% CI, 0.72, 2.43, *p* = 0.36; I² for OR meta-analysis = 72%), as presented in Fig. 4.

In a sensitivity analysis, we categorized patients as having mild or moderate MR, and severe MR, and the results also remained consistent (OR, 1.22, 95%CI, 0.38, 3.91, *p* = 0.74, I^2^ = 77).

## Discussion

The major findings of our systematic review with meta-analysis are: (1) there is no significant association between MR severity and the risk of TE in patients with non-valvular AF, and (2) the prevalence of either thrombotic or embolic events according to MR severity was not significantly different.

### Studies with similar findings

These findings align with two other studies that, due to data limitations, could not be incorporated into our meta-analysis [18, 19]. Bisson et al. included a total of 8,675 patients and compared the prevalence of TE in patients with and without MR over a follow-up period of 2.5 years, and found no significant differences [18]. Similarly, Philippart et al. conducted an analysis including 8,057 patients, comparing those with and without mitral valve disease, and found no significant difference in the incidence of TE [19]. Additionally, subgroup analyses focusing on patients with severe MR also failed to identify any significant differences. These studies support our conclusion that MR severity does not significantly correlate with the risk of thromboembolic occurrences.The reported overall incidence of stroke tended to be lower in our analysis compared to these two studies (7.8% for both studies [18, 19]).

### Key factors potentially contributing to the lack of difference

The lack of association between MR severity and thromboembolic risk might be explained by various factors. Baseline characteristics of our study population revealed that patients with moderate or more severe MR were older and had lower LVEF, both known as independent risk factors for TE [2, 12, 16, 20]. Moreover, both risk factors are included in the CHA_2_DS_2_-VASc score which was also significantly higher in patient with moderate or more severe MR. Despite differences in there thromboembolic factors between MR groups, the incidence of TE was observed to be similar. This congruence in TE rates, despite the heightened baseline risk in the moderate/severe MR cohort, suggests that either risk factors and any unmeasured protective elements achieve a balance, or that comparable anticoagulation use across groups effectively mitigates these underlying risk disparities. Moreover, the potential protective association of MR severity cannot be formally ruled out in view of the different populations comparison.

Another factor potentially influencing thrombogenesis is the direction of the MR jet and its inability to reach the LAA, where stasis and thrombus formation mainly occur. In particular, in the case of isolated flail/prolapse of the P3 scallop, the resulting MR jet is typically directed anterolaterally, potentially “washing” the LAA. Detailed data of this nature were not available in the studies that we assessed.

### Potentially associated risk factors for thromboembolic events

Some predictors of ischemic stroke in the context of AF have been suggested: a high CHA2DS2-VASc score [1, 16, 19, 21], pre-existing LAT, and low LAA emptying velocity [15, 16]. Van Laer et al. reported a reduction in LA spontaneous echo contrast (LASEC) in patients with severe MR, yet they did not document a similar finding with LAT, suggesting that the jet of MR may not extend to reach the LAA [15]. Among studies not fulfilling our inclusion criteria, several looked at the association of MR with LASEC. Fukada et al. identified a protective role of MR against LASEC but did not report the incidence of TE [22]. An interesting observation was reported by Nair et al. who conducted a study over a mean period of 34 months on a cohort of 84 high-risk patients with AF and LAT; they found no significant difference in the incidence of embolic events, reinforcing the idea that moderate or severe MR does not significantly alter the occurrence of embolic events even in this high risk group [23].

### Concept of atrial cardiopathy

Atrial cardiopathy has been postulated as a risk factor for stroke even in the absence of clinically apparent AF [24]. The underlying pathophysiological mechanism involves the development of atrial fibrosis and deformation of the LA, which results in increased stiffness and reduced contractility, thereby facilitating the process of thrombogenesis [2, 24]. A surrogate marker for atrial cardiomyopathy is atrial enlargement and abnormal strain of the LA on echocardiography [24]. In light of this, the ARCADIA trial investigated the relationship between atrial cardiopathy—based on electrocardiogram findings, NT-proBNP levels, and echocardiography—and stroke, by randomizing 1,015 patients with cryptogenic stroke to either apixaban or aspirin [25]. Despite the trial’s failure to prove a benefit of anticoagulation, only 13 patients met the criteria for LA enlargement, which limits the conclusions of this study. Indeed, a prior study reported a correlation of thromboembolic risk with the enlargement of the left atrium [12]. Consequently, AF might simply be a marker of underlying atrial dysfunction, a phenomenon insufficiently described in the included studies.

In our sensitivity analysis comparing patients with mild or moderate MR to those with severe MR [13, 15, 16], we found no significant difference with regards to TE, in contrast to other studies that demonstrated either an association with stasis in the LA or an effect on thrombus formation, particularly in patients with rheumatic MR [5, 6]. In addition to the need to standardize the definitions of MR, which may vary between studies, there is a necessity for better assessment and clarification of MR’s echocardiographic characteristics. This includes the direction of the MR jet and its interaction with the LAA, to deepen our comprehension of the theory behind LAA washout.

## Limitations

This meta-analysis is subject to potential limitations. We did not have access to individual patient data. As previously discussed, a better knowledge of these data would be key to understanding and interpreting the results. Additionally, it is pivotal to acknowledge that most studies were retrospective in design. The primary indication for TEE in these studies was external cardiac cardioversion in the AF context; therefore, whether patients who did not have TEE had LAT is unknown. Further, it is important to recognize that patients in the two groups differed significantly in baseline characteristics, limiting direct comparability. These differences could have an impact on the risk assessment for TE. Moreover, variability in methods for quantifying mitral regurgitation and definitions of thrombotic events, which were not always systematically assessed, may influence the reported incidence of events. Therefore, any conclusions drawn from this data must be considered with caution.

## Conclusions

Our analysis suggests that while MR plays a role in cardiac hemodynamics, its severity alone does not appear to be a decisive factor in determining thromboembolic risks in non-valvular AF patients. Future prospective studies with larger cohorts and standardized categorization of MR severity could provide more definitive answers.

## Supplementary Information

Below is the link to the electronic supplementary material.

Supplementary Material 1

## Data Availability

No datasets were generated or analysed during the current study.

## Abbreviations

AF: Atrial fibrillation
CI: Confidence Interval
LA: Left atrium
LAA: Left atrial appendage
LASEC: Left atrial spontaneous echo contrast
LAT: Left atrial thrombus
LVEF: Left ventricular ejection fraction
MR: Mitral regurgitation
NOS: Newcastle-Ottawa Scale
OR: Odds Ratio
PISA: Proximal Isovelocity Surface Area
SD: Standard Deviation
TE: Thromboembolic events
TEE: Transesophageal echocardiography
TIA: Transient ischemic attack
